# Functional validation of *AaCaM3* response to high temperature stress in *Amorphophallus albus*

**DOI:** 10.1186/s12870-024-05283-2

**Published:** 2024-06-28

**Authors:** Yi Niu, Zixuan Zhou, Zhenyu Yue, Xiaofei Zhang, Xuekuan Jiang, Lingyu Hu, Quanshuo Liu, Xu Zhang, Kun Dong

**Affiliations:** 1 Yibin Academy of Southwest University, Yibin, China; 2 College of Horticulture and Landscape Architecture, Southwest University, Chongqing, China; 3 Key Laboratory of Horticulture Science for Southern Mountainous Regions, Ministry of Education, Southwest University, Chongqing, China; 4 Chongqing SINO Konjac Biotechnology Co., Ltd, Chongqing, China; 5 Institute of Fuyuan Konjac, Yunnan Academy of Agricultural Sciences, Qujing, China

**Keywords:** *Amorphophallus Albus*, AaCaM3, Promoter-protein interaction, Heat stress

## Abstract

*Amorphophallus* is a perennial monocotyledonous herbaceous plant native to the southwestern region of China, widely used in various fields such as food processing, biomedicine and chemical agriculture. However, *Amorphophallus* is a typical thermolabile plant, and the continuous high temperature in summer have seriously affected the growth, development and economic yield of *Amorphophallus* in recent years. Calmodulin (CaM), a Ca^2+^ sensor ubiquitous in eukaryotes, is the most important multifunctional receptor protein in plant cells, which affects plant stress resistance by participating in the activities of a variety of signaling molecules. In this study, the key gene *AaCaM3* for the Ca^2+^-CaM regulatory pathway was obtained from *A. albus*, the sequence analysis confirmed that it is a typical calmodulin. The qRT-PCR results demonstrated that with the passage of heat treatment time, the expression of *AaCaM3* was significantly upregulated in *A. albus* leaves. Subcellular localization analysis revealed that AaCaM3 localized on the cytoplasm and nucleus. Meanwhile, heterologous transformation experiments have shown that *AaCaM3* can significantly improve the heat tolerance of *Arabidopsis* under heat stress. The promoter region of *AaCaM3* was sequenced 1,338 bp by FPNI-PCR and GUS staining assay showed that the promoter of *AaCaM3* was a high-temperature inducible promoter. Yeast one-hybrid analysis and Luciferase activity reporting system analysis showed that the *AaCaM3* promoter may interact with AaHSFA1, AaHSFA2c, AaHSP70, AaDREB2a and AaDREB2b. In conclusion, this study provides new ideas for further improving the signal transduction network of high-temperature stress in *Amorphophallus.*

**Supplementary Information:**

The online version contains supplementary material available at 10.1186/s12870-024-05283-2.

## Introduction

Konjac is a perennial monocotyledonous herb that originates from the understory of tropical rainforests. Konjac is not a specific species, it is a generic term for different species of *Amorphophallus* Blume in Araceae [1]. *Amorphophallu albus* (*A. albus*) is a variety of konjac native to southwestern China and it is widely cultivated in China because of the rich konjac glucomannan (KGM) content and excellent quality in the underground expanded bulbs [2, 3]. KGM is a soluble dietary fiber, which is widely used in food processing, biomedical, chemical and agricultural fields due to its good thickening, hydrophilic, film-forming and gelling properties. Research shows that konjac plays an important role in lowering sugar, preventing cardiovascular disease and slimming and has a very bright application prospects [4–6]. However, the world is confronted with the contradiction of reduced crop production and population explosion, the security of food supply is currently facing an unprecedented threat, and the excessive emission of greenhouse gases has further exacerbated global climate change [7]. High temperature stress is one of the most important environmental stresses that can cause severe and irreversible damage to plants [8, 9]. Konjac is a typical shade-tolerant plant, in the past few years, konjac has been frequently exposed to prolonged high temperatures due to climate change [10]. When the cultivation environment remains hot for a long time, konjac will grow slowly, the leaves will curl gradually, the reduced chlorophyll content leads to leaf discoloration or even wilting, appear symptoms of burn and heat damage, and even collapse and death, seriously affecting the growth, development and the yield of konjac [11].

In order to adapt to the deteriorating survival environment, plants have evolved to develop complex signaling networks that facilitate their survival and reproduction under unfavorable conditions by altering their metabolic levels to better adapt to the environment when they perceive variations in the temperature of their surroundings [12–14]. Calcium signaling has been confirmed in plant cells as a secondary messenger involved in signal transduction [15]. When plants experience abiotic stress or mechanical injury, the cytoplasmic Ca^2+^ concentration ([Ca^2+^]_cyt_) is elevated, and a high Ca^2+^ environment is detrimental to cells, at this time, plants can produce different Ca^2+^ binding proteins that induce the expression of genes related to the Ca^2+^-CaM signaling system, allowing Ca^2+^ binding proteins to sequester free Ca^2+^ in cells [16–18]. Calmodulin (CaM) is an important member of the Ca^2+^-CaM signaling pathway, and in plant cells CaM is the most important multifunctional receptor protein, which affects plant stress resistance by participating in the activities of various signaling molecules [19]. When the ([Ca^2+^]_cyt_) is elevated and calcium signals are generated, Ca^2+^ can bind to its receptor protein CaM, which can regulate the expression of related genes, induce the synthesis of heat stress proteins and regulate a series of physiological metabolic processes to enhance the adaptation of plants to high temperatures [20, 21]. Typical CaM proteins all contain multiple EF-hands structures with a three-dimensional molecular structure shaped like a dumbbell [22]. CaM has no enzymatic activity of its own, Ca^2+^ is reversibly bound to CaM by binding to the EF-hands on its structural domain to form a complex, and the originally spherical EF-hands structure is modified to an open conformation, which subsequently induces a conformational change in CaM and regulates their activity in a calcium-dependent manner by interacting with the target protein and inducing target enzyme activity [23–25].

To date, *CaM* genes have been identified in many plants and have been associated with plant growth and developmental processes and responses to high temperature stresses [26–28]. *AtCaM3* is thought to have a positive regulatory effect on heat stress tolerance in *Arabidopsis* [29]. Overexpression of the *AtCaM3* gene in *Arabidopsis* followed by heat treatment revealed that the transgenic plants were more heat tolerant compared to WT, whereas the *atcam3* mutant was less heat tolerant than WT, and Nitric oxide can enhance plant heat tolerance by activating *AtCaM3* to stimulate the expression of its downstream *HSF* and *HSP* genes [30, 31]. The expression levels of *NhDnaJ*, *NhVDAC* and *NhBCP* were significantly increased in *Neoporphyra haitanensis* under high temperature stress, and NhCaM1 can interact with them [32]. Heat treatment of *Arabidopsis* overexpressing *OsCaM1-1* showed that high temperature can induce the expression of heat stress-related genes such as *AtCBK3*, *AtPP7*, *AtHSFs*, and *AtHSPs* [33].

In this study, we cloned *AaCaM3* from *A. albus* and analyzed its subcellular localization, heterologous transformation, and gene regulation relationship. These results will further deepen our understanding of *AaCaM3* in response to high-temperature stress of *A. albus* and provide new ideas for further improvement of signal transduction network of konjac under high temperature stress.

## Materials and methods

### Plant materials and stress treatments

*A. albus* was selected as the experimental materials, and the materials were obtained from Xiema Konjac resource nursery (Xiema, Beibei, Chongqing, PR China). After disinfection, the materials were planted in flowerpots filled with a mixture garden soil and peat moss (2:1, V/V) in a greenhouse. *A. albus* were irrigated with Hoagland’s nutrient solution every 5 days. 70 days after planting, the *A. albus* leaves were fully extended, plants with normal growth, uniform plant height, and no pests and diseases were selected. Selected plants were transferred to the artificial climate incubator (25 ℃, 16 h light/8 h dark, light intensity of 12,000 lx) for pretreatment for two days. After pretreatment, the control group was grown at room temperature (25 ℃) and the experimental group samples were treated at 41 ℃ for high temperature. The leaves after 0 h, 1 h, 8 h and 24 h heat treatment were collected, all samples with three biological replicates were quickly freezed with liquid nitrogen and stored at -80 ℃.

### Molecular cloning of *CaM3* from *A. albus*

Total RNA of *A. albus* leaves was extracted using Quick RNA Islation Kit (Huayueyang, Beijing, China) from plant samples stored at − 80 ℃. cDNA of *A. albus* leaves was synthesized using PrimeScript™ RT reagent Kit (Takara, Dalian, China). gDNA of *A. albus* leaves was extracted using Polysacchardes & Polyphenolics-rich Plant Genomic DNA Kit (TIANGEN, Beijing, China). Sequences of *AaCaM3* were retrieved from the high-temperature stress RNA-seq library (not published yet) of *A. albus* leaves, and primers AaCaM3-F and AaCaM3-R (Table S1) for gene cloning were designed using the online website Primer3Plus. The target genes were amplified using Phanta®Max Super-Fidelity DNA Polymerase (Vazyme, Nanjing, China). The amplified fragments were cloned in pEASY®-Blunt Simple (TransGen, Beijing, China) and sequenced.

### DNA sequence analysis

The cloned *AaCaM3* gene sequence was nucleotide blast compared on the *A. konjac* genome in the NCBI database (National Center for Biotechnology Information). Using software such as DNAMAN and BioXM 2.7 for sequence alignment and amino acid sequence analysis. Protein sequences of CaM3 from other species were sought from NCBI, and the phylogenetic tree was constructed by using software MEGA X.

### *AaCaM3* expression analysis by quantitative real-time PCR (qRT-PCR)

Quantitative expression analysis were performed in PCR 8-Tube by using the Bio-Rad CFX96 Real-Time PCR system (Bio-Rad, USA). The reaction system was configured using the fluorescent quantitative PCR dye SYBR Green qPCR mix (Biosharp, Hefei, China), and the most stable internal reference genes *EIF4A* under heat stress were used as the reference gene [2]. Three biological replicates and three technical replicates were set for each sample and three mock-free controls were set. The comparative CT method (2^−ΔΔCt^) was used to calculate the relative expression amount of the target gene.

### Subcellular localization of AaCaM3

In order to observe the subcellular localization of AaCaM3 protein, we generated a pCAMBIA1300- AaCaM3-GFP vector. Using vector pEASY-Blunt- AaCaM3 as template, the open reading frame (ORF) of *AaCaM3* without a termination codon was amplified by CaM3-1300-F-Xba I and CaM3-1300-R-Kpn I primers (Table S1). The obtained vector pCAMBIA1300- AaCaM3-GFP and the control empty vector pCAMBIA1300-GFP were separately transformed into tobacco epidermal cells by transduction of *Agrobacterium tumefaciens* strain LBA4404. The fluorescence signal in tobacco epidermal cells was observed using a laser scanning confocal microscopy of Zeiss.

### Transformation of *AaCaM3* into *A. thaliana*

The overexpression vector AaCaM3-pBin35SRed3 was constructed to transform into *Arabidopsis thaliana* by *A. tumefaciens*-mediated inflorescence infection method (Fig. S2). The flowering pods of *A. thaliana* growing in the same condition were cut off, and water them thoroughly, inflorescence infection 1–2 min. All harvested *Arabidopsis* seeds were collected, and the emitting seeds were screened under the LUYOR-3415RG excitation light source and then homozygote transgenic *Arabidopsis* were obtained after three generations of continuous screening.

### Thermotolerance test of transgenic *A. thaliana* plants

For thermotolerance evaluation on AaCaM3-overexpression *Arabidopsis*, WT and transgenic *Arabidopsis* seedlings grown on MS medium for 14 d were exposed to 41 ℃/1 h, 41 ℃/6 h and 41 ℃/12 h, and then phenotypic analysis survival analysis were performed after recovery for 3 days.

### Cloning and analysis of *AaCaM3* promoter

The *AaCaM3* promoter was cloned from gDNA of *A. albus* leaves by FPNI-PCR [34]. Three rounds of nested FPNI-PCR reactions were performed to amplify the sequences in the promoter region by upstream degenerate primers FP1-9, FSP1, FSP2 and downstream primers AaCaM3-SP1, AaCaM3-SP 2, AaCaM3-SP3 (Table S1). Prediction of *cis*-acting elements for the *AaCaM3* promoter by online sites PlantCare, and PlantPAN3.0.

In order to explore the characteristics of the *AaCaM3* promoter, we generated a pMDC162- AaCaM3 vector. Using vector pEASY-Blunt-AaCaM3 as template, the ORF of *AaCaM3* with *Kpn* I and *Asc* I digestion sites was amplified using prCaM3-162-F (*Kpn* I) and prCaM3-162-R (*Asc* I) primers (Table S1). The obtained vector pMDC162-AaCaM3 was transformed into tobacco leaves by transduction of *A. tumefaciens* strain GV3101. Cultivate in the dark for 36 h, and then place them at 25 ℃ and 41 ℃, respectively. Tobacco plants cultured at 25 ℃ were used as the control group, and plants treated at 41 ℃ were used as the experimental group. After treatment for 1 h, 8 h, and 24 h, tobacco leaves were taken. Use the GUS staining kit (Coolaber, Beijing, China) to stain the tissue of tobacco leaves.

### Yeast one-hybrid assay

Construction of Y1H expression vector prAaCaM3-pAbAi using primer design software CE Design V1.04. Using vector pEASY-Blunt-prAaCaM3 as template, the *prAaCaM3* with *Kpn* I and *Xho* I digestion sites was amplified using prAaCaM3-pAbAi-F (*Kpn* I) and prAaCaM3-pAbAi-R (*Xho* I) primers (Table S1). The Y1HGold receptor state was produced by the Y1HGold solution stored at -80 ℃and drawing a single line on 1×YPDA (1× Yeast Extract Peptone Dextrose Adenine Medium) solid medium for activation. The recombinant plasmid prAaCaM3-pAbAi was digested with *Bst*B I restriction endonuclease and the linearized recombinant plasmid was transformed into the Y1HGold receptor state. The bacterial solution was named Y1HGold (prAaCaM3-pAbAi) and stored at -80 ℃. The diluted Y1HGold(pAbAi-prAaCaM3) bacterial solution was coated on SD/-Ura medium containing different concentrations (0-500 ng/mL) of AbA and cultured at 28 ℃ for 3–5 days to screen for AbA resistance concentrations.

The Y1HGold (prAaCaM3-pAbAi) receptor state was produced by the Y1HGold (prAaCaM3-pAbAi) solution stored at -80 ℃ on 1×YPDA medium. The constructed pGADT7 recombinant plasmids pGADT7-AaHSFA1, pGADT7-AaHSFA2c, pGADT7-AaHSP70, pGADT7-AaDREB2a and pGADT7-AaDREB2b were transformed into Y1HGold (prAaCaM3-pAbAi) to detect the interaction of *prAaCaM3* with the protein. The potential physical interactions between promoter-proteins were evaluated by screening the yeast transformants on selective mediums (SD/-Leu/AbA^300^, SD/-Leu medium with 300 ng/mL concentration of AbA) at 28 ℃ for 3–5 days.

### Dual-luciferase reporter assay

We constructed recombinant plasmids pGreenII0800-LUC-prAaCaM3, pGreenII62-SK-AaHSFA1, pGreenII62-SK-AaHSFA2c, pGreenII62-SK-AaHSP70, pGreenII62-SK-AaDREB2a, pGreenII62-SK-AaDREB2b and transformed them into GV3101-pSoup receptor state in order to verify the results of Yeast One-hybrid. The recombinant plasmids containing the promoter and the recombinant plasmids containing the gene were mixed separately at a volume ratio of 1:2 and then placed for 2 h at room temperature and protected from light, then injected into tobacco leaves. GV3101-pSoup (pGreenII0800-LUC-prAaCaM3) was combined with GV3101-pSoup (pGreenII62-SK-AaHSFA1), GV3101-pSoup (pGreenII62-SK-AaHSFA2c), GV3101-pSoup (pGreenII62-SK-AaHSP70), GV3101-pSoup (pGreenII62-SK-AaDREB2a) and GV3101-pSoup (pGreenII62-SK-AaDREB2b) mixed injected tobacco leaves as experimental groups, while GV3101-pSoup (pGreenII0800-LUC-prAaCaM3) and GV3101-pSoup (pGreenII62-SK) mixed injected tobacco leaves as the control group.

After 48 h dark culture, the injected leaves were removed and 0.2 mg/mL of D-Luciferin potassium salt was applied to the surface of the tobacco leaves. It was placed in dark for 7–8 min, and then the Vilber chemiluminescence and in vivo imaging system was used to capture the chemiluminescence results and calculate the relative luminescence intensity using Image J.

## Results

### Cloning and sequence analysis of *AaCaM3*

**Fig. 1:**
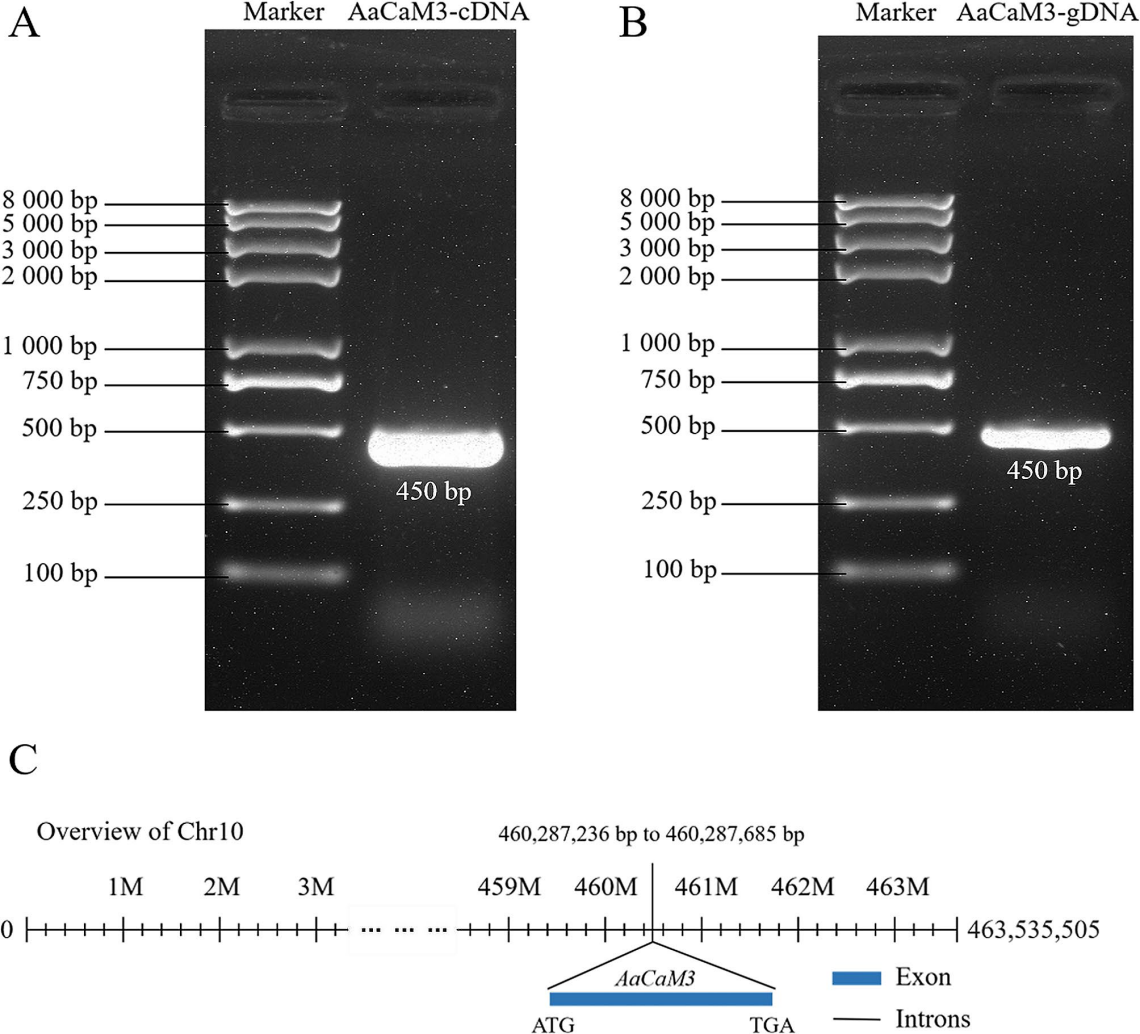
PCR amplification of *AaCaM3* gene from *A. albus* on agarose gel and its location in the genome of *A.konjac*. **A**: Cloned using cDNA as template; **B**: Clones cloned using gDNA as template; **C**: *AaCaM3* gene location in the genome of *A.konjac*. Excess gel is cropped in the image

The coding sequence (CDS) of *AaCaM3* gene was obtained by PCR amplification using cDNA of *A. albus* leaves as template. The full-length CDS sequence of *AaCaM3* is 450 bp, encoding a protein of 149 amino acids (Fig. 1-A). The predicted molecular mass of AaCaM3 protein is about 16.996 kDa, with an isoelectric point (pI) of 4.34 and an instability coefficient of 28.20, which is a negatively charged and stable protein. The gDNA sequence of *AaCaM3* was obtained by PCR cloning using the gDNA of *A. albus* leaves as a template, and the sequence of *AaCaM3* from gDNA is 450 bp (Fig. 1-B). The sequences of the *AaCaM3* gene obtained by cloning from cDNA and gDNA as templates, respectively, were compared and found to be identical, indicating that no introns exist in the *AaCaM3* gene. The *AaCaM3* gene sequence was blast compared on the *A.konjac* genome and found to be highly concordant with the sequence at 460,287,236 bp to 460,287,685 bp on chromosome 10, with 99.56% sequence similarity (Fig. 1-C).

**Fig. 2:**
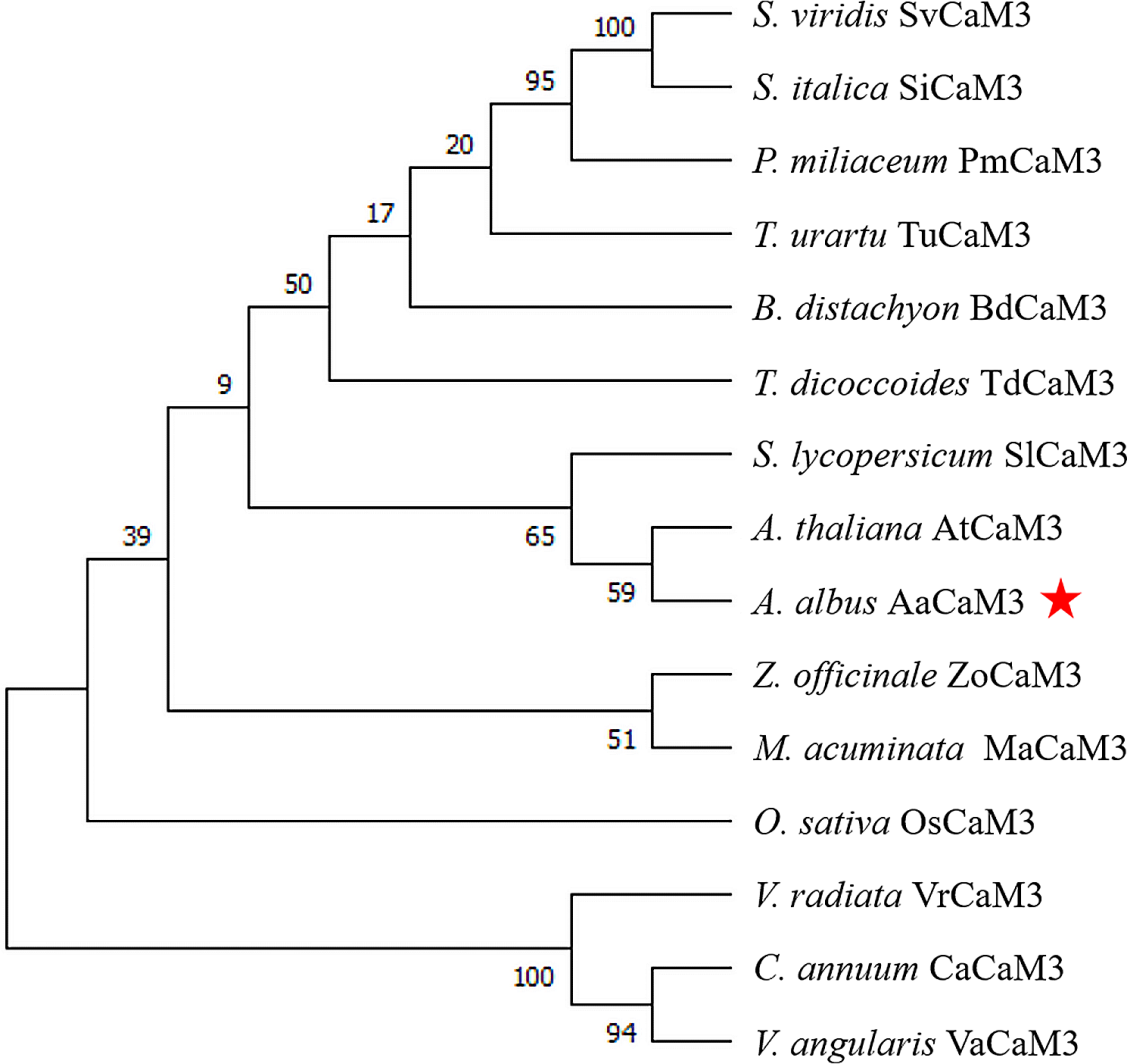
Phylogenetic analysis of AaCaM3 (red star marker). SvCaM3(XP_034593254.1); SiCaM3(XP_004970312.1); PmCaM3(RLM91395.1); TuCaM3(EMS58580.1); BdCaM3(XP_003567308.1); TdCaM3(XP_037412708.1); SlCaM3(NP_001308423.1); AtCaM3(NP_191239.1); ZoCaM3(XP_042384124.1); MaCaM3(XP_009417893.1); OsCaM3(XP_015621336.1); VrCaM3(XP_014495828.1); CaCaM3(KAF3663584.1); VaCaM3(KAG2403671.1)

The CDS-encoded protein sequences of the *AaCaM3* gene was queried according to NCBI, and we found the typical features of calmodulin containing multiple EF-hand modules and Ca^2+^ binding sites for this protein. The amino acid sequence of AaCaM3 was then compared with other known members of the CaM3 protein obtained from NCBI, such as MaCaM3, OsCaM3, ZoCaM3, SlCaM3 and AtCaM3. A phylogenetic tree containing the AaCaM3 protein was constructed using MEGA X software. As shown in the phylogenetic tree, AaCaM3 is closer to SlCaM3 and AtCaM3. These results demonstrate the accuracy of *AaCaM3* cloning in this study (Fig. 2). Besides, *AtCaM3* is thought to have a positive regulatory effect on heat stress tolerance in *Arabidopsis* [29–31], we speculate that *AaCaM3* has similar function in *A. albus.*

**Fig. 3:**
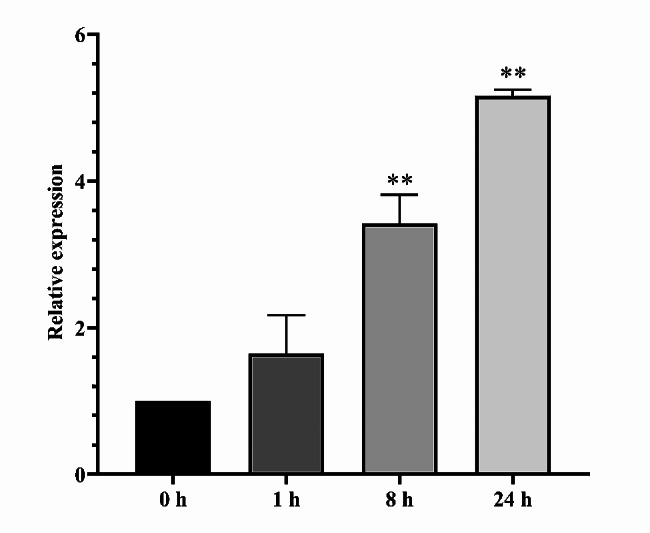
Relative expression of *AaCaM3* from *A. albus* at different time after heat treatment at 41 ℃. Bars within each panel are significant differences at * *P* < 0.05, ** *P* < 0.01 according to the Turkey’s test

To investigate the expression pattern of *AaCaM3* gene, we analyzed the expression levels of *A. albus* leaves at different time after heat treatment at 41 ℃ using qRT-PCR. The results showed that *AaCaM3* in leaves was sensitive to the induction of heat stress, and its expression showed a continuous upward trend with increasing heat treatment time (Fig. 3). These results indicate that the expression levels of *AaCaM3* differed at different heat treatment times, which implies that transcriptional regulation of the *AaCaM3* gene plays an important role in the heat tolerance.

### Subcellular localization

**Fig. 4:**
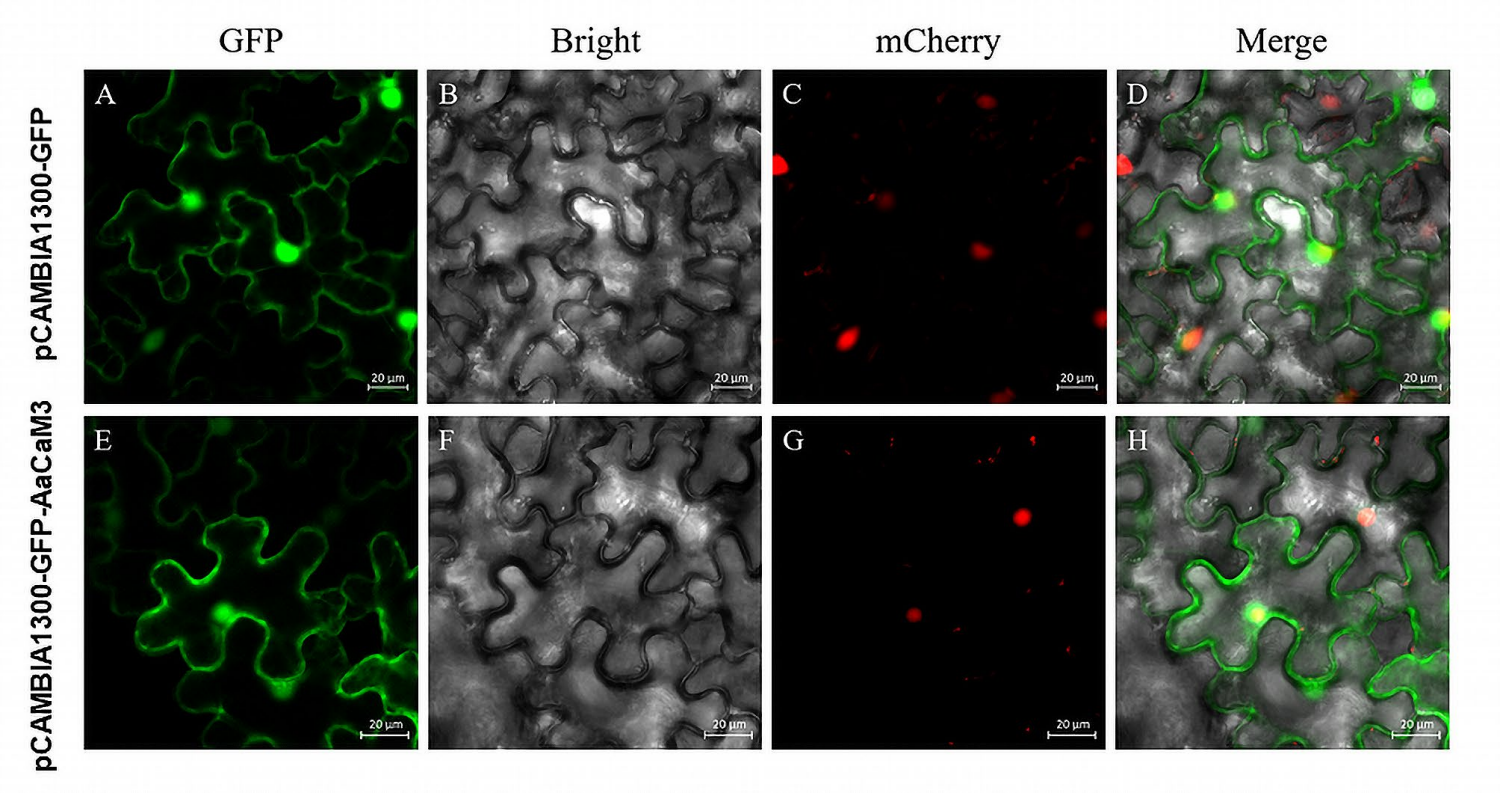
Subcellular localization analysis of AaCaM3 in tobacco leaves. mCherry were used as markers for nucleus. **A-D** are subcellular localization of pCAMBIA1300-GFP; **E-H** are subcellular localization of AaCaM3 protein

The subcellular localization online prediction site TargetP1.1 predicts AaCaM3 localization in the cytoplasm and nucleus of cells. To research the subcellular localization of AaCaM3, restriction site analysis of *AaCaM3* gene and pCAMBIA1300-GFP vector was performed using BioXM 2.7 software based on the CDS sequence of *AaCaM3* gene of *A. albus*. The pCAMBIA1300-AaCaM3-GFP subcellular localization expression vector was constructed using *Xba* I and *Kpn* I digestion sites. Tobacco leaves injected with pCAMBIA1300-GFP were used as the control group, and tobacco leaves injected with AaCaM3-pCAMBIA1300-GFP were used as the experimental group. Subcellular localization of AaCaM3 was successfully determined by transient expression of tobacco leaf epidermal cells. Under laser confocal microscopy of the GFP signaling pathway (488 nm), the AaCaM3-GFP recombinant protein was found to be mainly localized to the cytoplasm and nucleus (Fig. 4).

### Overexpression of *AaCaM3* increases heat stress tolerance in transgenic *Arabidopsis*

**Fig. 5:**
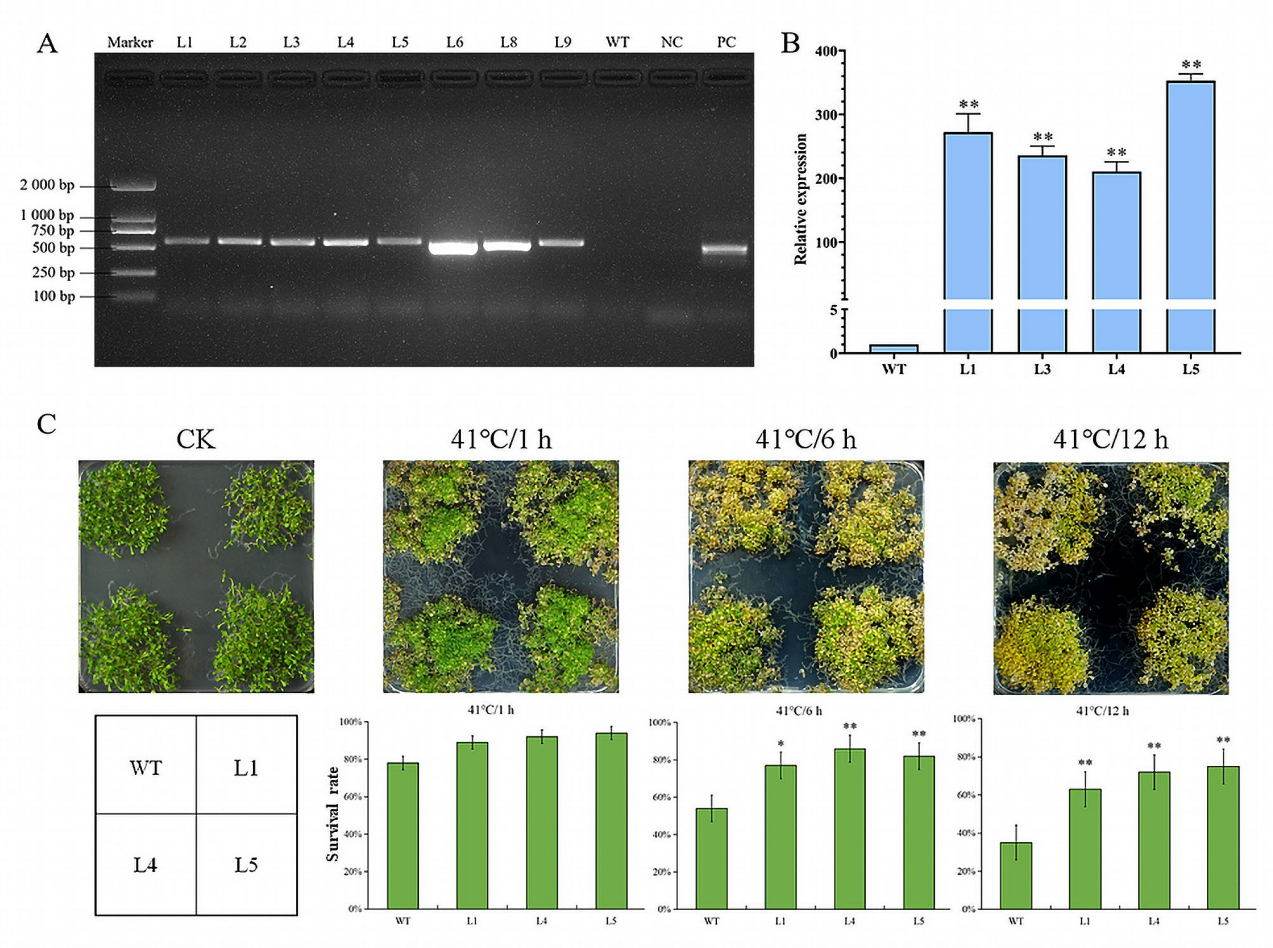
Overexpression of *AaCaM3* increases heat stress tolerance in transgenic *Arabidopsis*. **A**: Electrophoresis map of transgenic *Arabidopsis* DNA detection (L1, L2, L3, L4, L5, L6, L8, L9: transgenic lines 1, 2, 3, 4, 5, 6, 8, 9; WT: Wild-type *Arabidopsis*; NC: without template; PC: AaCaM3-pBin35SRed3 recombinant plasmid); **B**: Expression level of *AaCaM3* in different transgenic *Arabidopsis* plants (WT: Wild type; L1, L3, L4, L5: Transfer of *AaCaM3* gene to *Arabidopsis*;); **C**: Survival rate of wild type *Arabidopsis* and transgenic *Arabidopsis* after heat treatment (CK: Seeds exposed to 26 ℃ as a control. WT: Wild-type *Arabidopsis*; L1, L4, L5: Transgenic lines). Bars within each panel are significant differences at * *P* < 0.05, ** *P* < 0.01 according to the Turkey’s test

To further investigate the role and gene biological functions of *AaCaM3* in high temperature stress of the *A. albus*, we constructed the overexpression vector pBin35SRed3-AaCaM3 which was transferred into *Agrobacterium* GV3101 and heterologously transformed *Arabidopsis* by floral dip. After successive multi-generation screens, we successfully generated eight independent AaCaM3-overexpression transgenic *Arabidopsis* lines. The expression of *AaCaM3* in transgenic *Arabidopsis* was detected in all pBin35SRed3-AaCaM3 transgenic lines but not in the WT plants (Fig. 5-A). Leaf RNA of L1, L3, L4 and L5 transgenic *Arabidopsis* for *AaCaM3* gene was extracted and the expression levels of *AaCaM3* gene in transgenic strains L1, L3, L4 and L5 were detected by qRT-PCR successfully. The results showed that the *AaCaM3* gene was overexpressed in different transgenic *Arabidopsis* strains compared with the WT, indicating that the transgenic *Arabidopsis* strains L1, L3, L4 and L5 were indeed *Arabidopsis* transgenic plants with *AaCaM3* and could be tested in follow-up experiments (Fig. 5-B).

To further verify whether transgenic lines of *AaCaM3* could improve the tolerance of transgenic *Arabidopsis* plants with high temperature stress, we performed a simulated heat treatment of transgenic lines for phenotypic characterization. Since we didn’t harvest enough seeds in transgenic line L3, transgenic lines L1, L4 and L5 were chosen for follow-up experiments. The results showed that there is no significant difference between transgenic plants and WT in seed germination, seedling growth, and development under normal growth conditions, and the plants were high temperature treated at 41 ℃ for 1 h, 6 h and 12 h, respectively, and then resumed growth under normal conditions for 3 d. It was found that the survival rate of transgenic and WT *Arabidopsis* seedlings was almost unaffected after 1 h of treatment at 41 ℃, and there was little difference in plant survival rate. After 6 h of treatment at 41 ℃, the difference between transgenic and WT seedlings began to appear, and both began to wilt and die, the survival rate of transgenic plants was significantly higher than that of WT. After 12 h of treatment at 41 ℃, both transgenic and WT seedlings wilted and died in large numbers, but the survival rate of transgenic plants was greater than 60%, which was significantly higher than that of WT (Fig. 5-C).

### Promoter cloning and analysis of *AaCaM3*

Three rounds of FPNI-PCR were using to clone the promoter region sequence of *AaCaM3*, and the promoter region sequence 1338 bp of *AaCaM3* was obtained. The cloned promoter region sequence was compared with the *A.konjac* genome, and it was found that the sequence mapped to chromosome 10 from 460,285,896 bp to 460,287,235 bp with 99.11% similarity, while the *AaCaM3* gene mapped to chromosome 10 from 460,287,236 bp to 460,287,685 bp, indicating that the sequence obtained by FPNI-PCR was indeed the promoter region of *AaCaM3*.

**Fig. 6:**
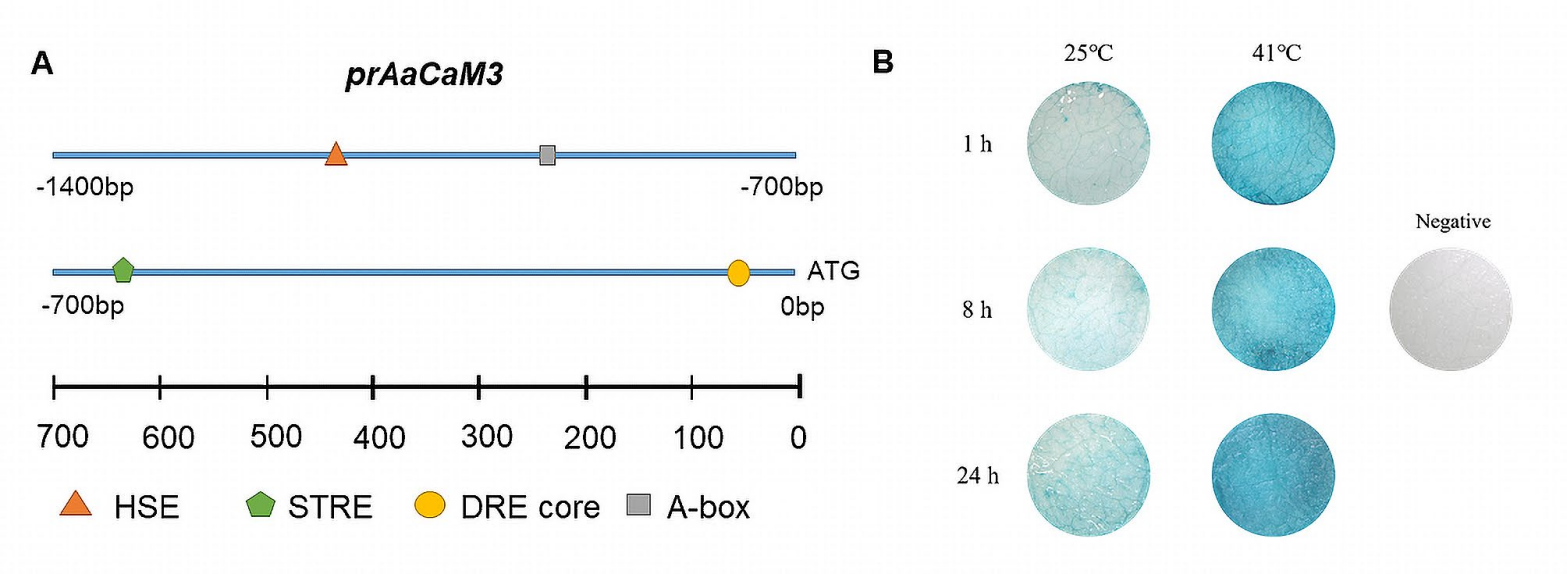
Analysis of *cis*-acting elements of *AaCaM3* promoter and GUS staining in tobacco leaves. **A**. Prediction of key *cis*-acting elements on *prAaCaM3* sequence; **B**: GUS staining of GV3101(prAaCaM3-pMDC162) in tobacco leaves

The *cis*-element prediction of *prAaCaM3* by online websites PlantCare and PlantPAN3.0 showed that the *AaCaM3* promoter has multiple promoter core elements such as CAAT-box and TATA-box, stress response elements such as DRE core, TC-rich repeats, STRE and HSE elements, the regulatory characteristics are complex (Fig. 6-A; Fig.S3). These results suggest that the *AaCaM3* gene may play an important role in abiotic stresses in *A. albus*.

Using GUS expression vector to explore the *AaCaM3* promoter activity and heat-inducible properties. GV3101 (prAaCaM3-pMDC162) was injected into tobacco leaves and incubated for 36 h at 25 ℃ and then placed at 25 ℃ (control group) and 41 ℃ (experimental group), respectively. Tobacco leaves from the experimental and control groups were sampled at 1 h, 8 h and 24 h for GUS staining to detect the expression of *GUS* genes. The GUS staining results showed that blue spots appeared in the control leaves but were lighter in color and smaller in area, indicating that *prAaCaM3* has promoter activity to drive small amounts of downstream *GUS* gene expression. Blue spots were also present on the leaves of the experimental group after the high temperature stress treatment at 41 ℃, but the color was darker and larger in the experimental group compared with the control group, indicating that high temperature can induce *prAaCaM3* to drive a large amount of downstream *GUS* gene expression, suggesting that *prAaCaM3* have heat-inducible (Fig. 6-B).

### Detection of *AaCaM3* promoter-protein interactions by yeast one hybrid (Y1H) and luciferase reporter system activity assay

**Fig. 7:**
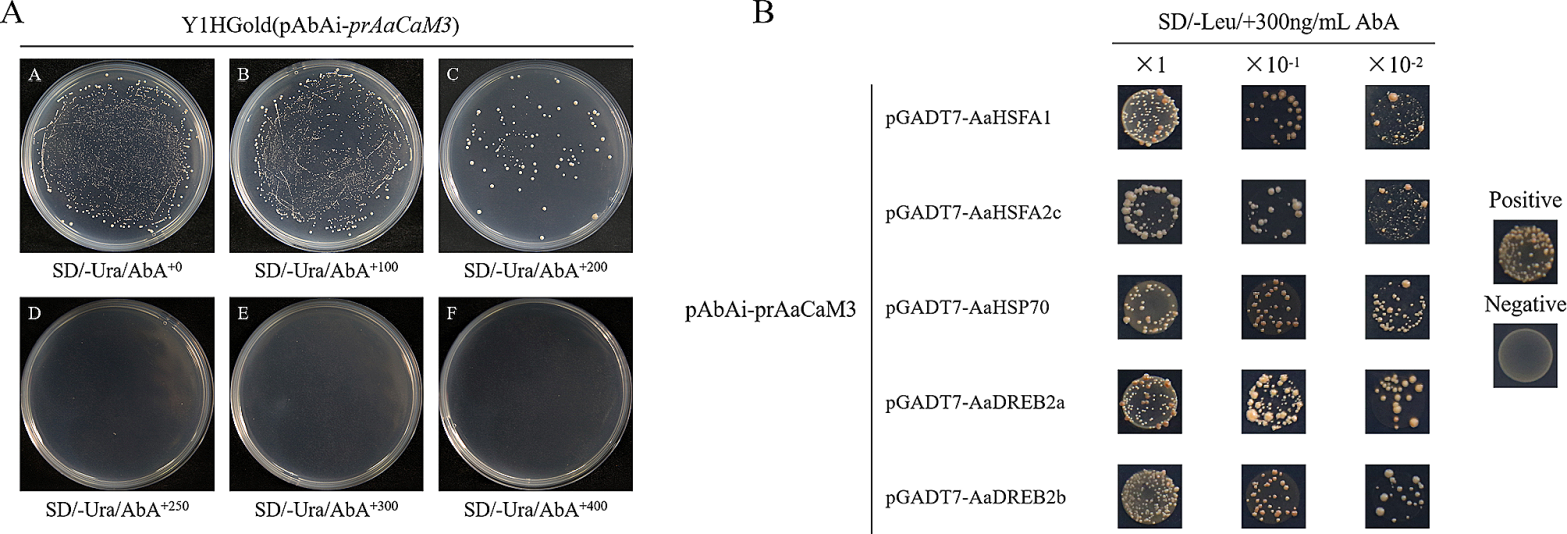
Detection of *AaCaM3* promoter-protein interactions by Y1H. **A**. Screening of ABA substrate concentration for Y1Hgold (pAbAi- prAaCaM3); **B**. Verification of promoter-protein interactions by yeast one-hybrid

Construction of Y1H expression vector prAaCaM3-pAbAi and the Y1HGold receptor state was produced by the Y1HGold solution stored at -80 ℃. The linearized recombinant plasmid prAaCaM3-pAbAi with *Bst*B I was transformed into the Y1HGold receptor state, and was named Y1HGold (prAaCaM3-pAbAi) and stored at -80 ℃. The diluted Y1HGold (pAbAi-prAaCaM3) bacterial solution was coated on SD/-Ura medium containing different concentrations of AbA for screening of AbA resistance concentration respectively. The results showed that Y1Hgold (pAbAi-prAaCaM3) could grow normally on SD/-Ura plates, indicating that the recombinant yeast strain Y1HGold(pAbAi-prAaCaM3) could be tested normally in subsequent experiments. Meanwhile, Y1HGold(pAbAi-prAaCaM3) could also grow yeast spots on plates with AbA concentrations of 100 ng/mL and 200 ng/mL, but the growth number and density gradually decreased, and when the AbA concentration in SD/-Ura plates reached 300 ng/mL, yeast could not grow, and yeast could not grow when the AbA concentration continued to be increased. The yeast could grow sporadically when the concentration of AbA in the plate was 250 ng/mL, so 300 ng/mL was chosen as the optimum concentration of AbA resistance in the subsequent test (Fig. 7-A).

The *AaCaM3* promoter has typical stress response elements such as DRE core elements and HSE elements (Fig. 6-A). We speculate that *AaCaM3* may be at a key position in the high tempertature stress regulatory network of *A. albus* and is associated with heat stress related genes such as *AaHSFs* and *AaHSPs* previously studied in the laboratory. Besides, it has also been reported that DREB proteins play an important regulatory role in plant response to high temperature stress, we identified two typical *DREB* transcription factors *DREB2a* and *DREB2b* in our high tempertature stress transcriptome of konjac, and speculated that they might regulate the expression of *AaCaM3*.

The Y1HGold (prAaCaM3-pAbAi) receptor state was produced by the Y1HGold (prAaCaM3-pAbAi) solution stored at -80 ℃ into a sterile inoculation loop and drawing a single line on 1×YPDA medium. The constructed pGADT7 recombinant plasmids pGADT7-AaHSFA1, pGADT7-AaHSFA2c, pGADT7-AaHSP70, pGADT7-AaDREB2a and pGADT7-AaDREB2b were transformed into Y1HGold (prAaCaM3-pAbAi) to detect the interaction of *prAaCaM3* with the protein. The results showed that the above three different concentrations (original concentration, ×0.1 concentration and ×0.01 concentration) of the transformed recombinant plasmid could grow on SD/-Leu/AbA^300^, and positive control grew normally while negative control failed to grow, suggesting that AaHSFA1, AaHSFA2c, AaHSP70, AaDREB2a and AaDREB2b may interact with the promoter of AaCaM3 to activate downstream reporter genes (Fig. 7-B).

**Fig. 8:**
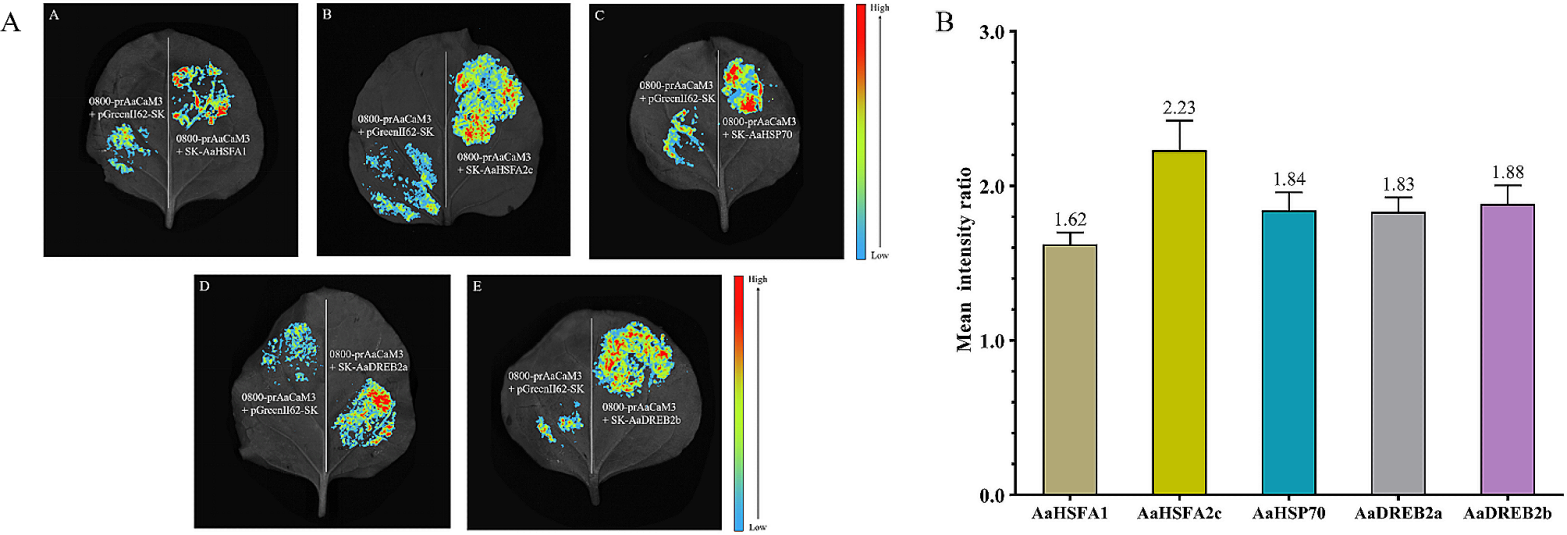
Detection of *AaCaM3* promoter-protein interactions by luciferase reporter system activity assay. **A**. Identification of promoter-protein interactions by luciferase reporter assay (In the above pictures, the left side of the tobacco leaf is the control group, and the right side of the leaf is the experimental group); **B**. Mean intensity ratio of experimental group and control group

The results were further validated with a luciferase reporter system activity assay, and results showed that the fluorescence intensity was stronger at the site of the experimental injection group compared to the control group (Fig. 8-A). Using Image J to calculate the mean luminescence intensity, the mean luminescence intensity of 0800-prAaCaM3 with SK-AaHSFA1, SK-AaHSFA2c, SK-AaHSP70, SK-AaDREB2a and SK-AaDREB2b was 1.62, 2.23, 1.84, 1.83 and 1.88 fold higher than the control group (Fig. 8-B). The calculated results showed that the mean luminescence intensity of the experimental groups were all significantly stronger than that of the control group. The above results suggest that AaHSFA1, AaHSFA2c, AaHSP70, AaDREB2a and AaDREB2b may interact with *prAaCaM3* and promote the expression of *AaCaM3*.

## Discussion

As a key gene in the Ca^2+^-CaM regulatory pathway, the expression of *AaCaM3* was significantly up-regulated with increasing time of high temperature stress treatment. The sequence of *AaCaM3* gene obtained from *A. albus* was 450 bp, encoding 149 amino acids (aa) and the alignment analysis showed that *AaCaM3* gene had no intron and structure domain analysis revealed that it contains several EF-hand motifs and Ca^2+^ binding sites, which is similar to other species. A comprehensive analysis of the phylogeny, conserved motifs and gene structure of *HvCaMs/CMLs* revealed that *HvCaMs/CMLs* were classified into nine groups. Five *HvCaMs* of barley forming a Group I, all of these *HvCaMs* having no introns and the same conserved motif [35]. The qRT-PCR showed that the expression of *AaCaM3* gene was up-regulated after high temperature stress, which was also consistent with the experimental results of other species. The expression of *AtCaMs* was measured after heat treatment in *Arabidopsis*, and by comparing the expression changes of *AtCaMs* after heat treatment, it was found that the expression of *AtCaM3* was significantly increased in all *AtCaMs* [29]. Five *LlCaMs* genes were isolated from lily, in which the expression of *LlCaM3* gene was positively correlated with lily heat tolerance [36]. It can be speculated that *AaCaM3* plays an important role in the resistance of konjac to heat stress.

Subcellular localization of the researched proteins helps us to understand their functions, and currently transient expression of recombinant fluorescent proteins in plants is a common method to detect the localization of target proteins in plant cells because transient expression techniques are not affected by gene silencing and have more stable expression efficiency and higher translation rates [37–39]. In this study, subcellular localization analysis revealed that AaCaM3 localized on the cytoplasm and nucleus, the result is similar to what predicted by the online prediction software TargetP1.1 and is also largely similar to the localization of CaM3 in other plants. The 35 S-LlCaM3-GFP construct was transformed into onion to determine the subcellular localization of LlCaM3 protein. Fusion protein of LlCaM3-GFP was detected in both the cytoplasm and nucleus and the results indicated that LlCaM3 was a cytoplasm and nucleus protein [36]. However, the localization of CaM3 is not the same in all species, for example, FvCaM3 of strawberry is specifically localized in the nucleus in protoplasts [40]. It is therefore speculated that the localization of CaM3 may have species differences.

According to reports from other species, overexpression of the *CaM3* gene significantly improved the heat tolerance of transgenic plants. Overexpression of the *AtCaM3* gene in *Arabidopsis* can significantly improve the heat tolerance of *Arabidopsis*, whereas the T-DNA inserted *atcam3* mutant has a lower heat tolerance than WT, and re-transferring *AtCaM3* into the *atcam3* mutant can recover its reduced heat tolerance [30]. The *LlCaM3* gene of lily was transferred into *Arabidopsis* and treated with high temperature stress. The results showed that the survival rate of the transgenic strain was 27–41% higher than WT strain, and the relative expression of *AtHsfA1a* and *AtHsp18.2* genes in the transgenic strain was 60% and 110% higher than WT plants, respectively, indicating that overexpression of *LlCaM3* in *Arabidopsis* can promote the expression of *AtHsp18.2*, *AtHsfA1a* and other high-temperature stress genes [36]. In summary, it can be concluded that the *AaCaM3* gene can enhance the heat tolerance of *A. albus* and play an important role in the regulation of high temperature stress.

The *cis*-regulatory elements on the promoter can activate or repress the expression of downstream genes by binding to transcription factors in transcriptional regulation, so research on the promoter of the *AaCaM3* gene helps us understand the mechanism of the Ca^2+^-CaM regulatory pathway and the upstream and downstream relationships of the gene [41, 42]. The promoter region sequence 1338 bp of *AaCaM3* was obtained by FPNI-PCR, and several *cis*-regulatory elements related to plant adversity and high temperature stress response element HSE were found on the promoter sequence, indicating its complex regulatory properties [34]. Inducible promoters are those that can drive the expression of downstream exogenous genes under specific environments [43, 44]. The *AaCaM3* promoter cloned in this experiment was able to drive the massive expression of downstream *GUS* gene in plant green tissues under high temperature conditions, indicating that *AaCaM3* is a heat-inducible promoter that can enhance the resistance of *A. albus* under high temperature stress, making it promising for application in resistance genetic engineering and genetic improvement.

Because *prAaCaM3* contains several *cis*-regulatory elements associated with plant adversity, especially the high-temperature stress response element HSE can interact with high-temperature stress-associated proteins. The results of previous studies indicate that *AaHSFA1* and *AaHSP70* play important roles in the regulatory network of high temperature stress in *A. albus* [11]. The results of Y1H and luciferase reporter system activity assay suggest that AaHSFA1, AaHSFA2c, AaHSP70, AaDREB2a and AaDREB2b can bind to the *AaCaM3* promoter and may promote the expression of *AaCaM3*. It has also been reported that DREB proteins play an important regulatory role in plant response to high temperature stress. *LlWRKY22* is a high temperature response regulator of Lily, can actively participate in the heat tolerance of lily by activating the expression of itself and *LlDREB2B*, suggesting that *LlDREB2B* is a key gene in the regulation of heat tolerance in lily [45].

In summary, in this study, we first studied the key gene of Ca^2+^-CaM regulatory pathway *AaCaM3* of *A. albus* in response to heat stress. Analysis of the role of *AaCaM3* gene in high temperature stress and the related molecular regulatory mechanisms involved in *A. albus* by bioinformatics analysis, subcellular localization, heterologous transgenesis, yeast one-hybrid and luciferase reporter system activity assay. These results are expected to initially clarify the mechanism of action of the key gene *AaCaM3* in the Ca^2+^-CaM regulatory pathway of *A. albus* in response to high-temperature adversity, providing new ideas to further improve the study of the signal transduction network of high-temperature stress in konjac, and also providing a basis for the comprehensive elucidation of the mechanism of plant heat tolerance formation.

### Electronic supplementary material

Below is the link to the electronic supplementary material.

Supplementary Material 1

Supplementary Material 2

## Data Availability

The datasets used and/or analyzed during current study are available from corresponding author on reasonable request.
