# Supplementary material for: Functional validation of AaCaM3 response to high temperature stress in Amorphophallus albus
Source: BMC Plant Biol. 2024 Jun 28;24:615. doi: 10.1186/s12870-024-05283-2 (PMC11212397; doi:10.1186/s12870-024-05283-2)
Supplement: Supplementary file 1 — Supplementary Material 1 [file 12870_2024_5283_MOESM1_ESM.pdf]

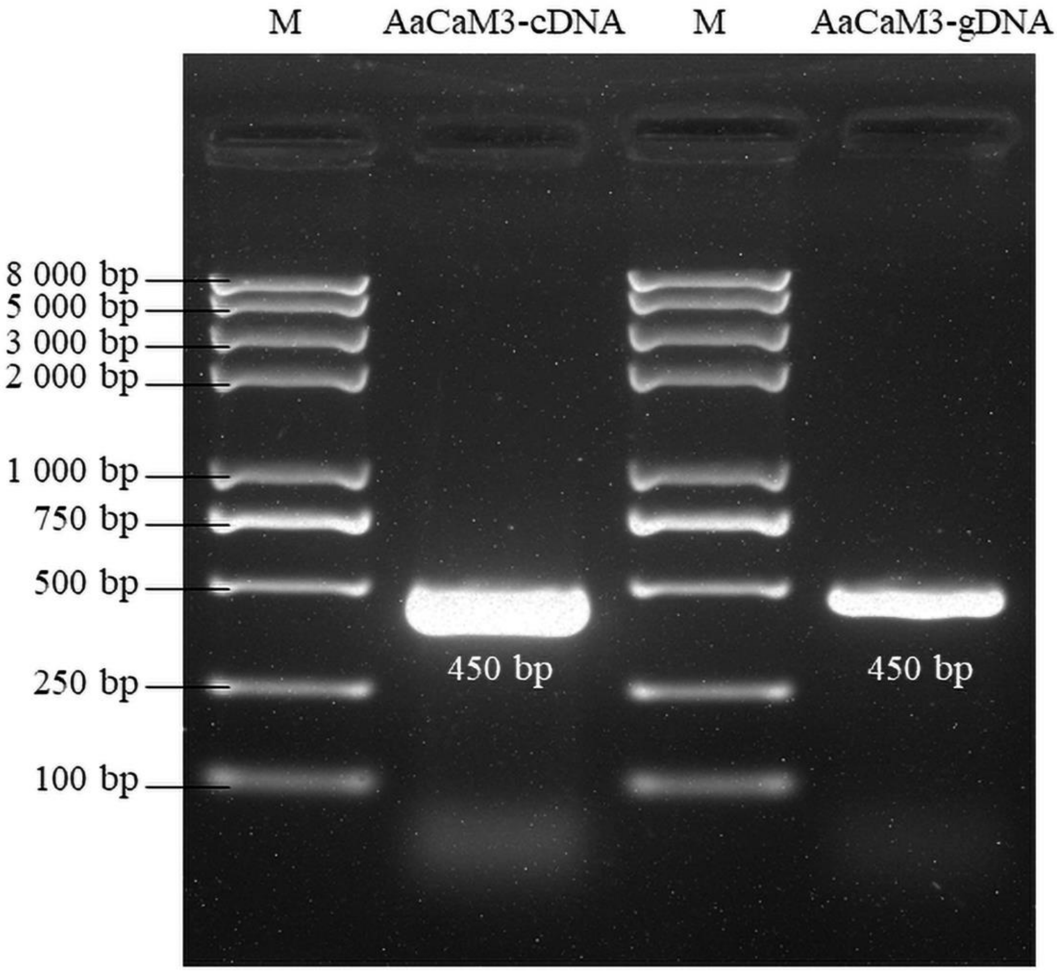

Figure S1 PCR results of AaCaM3 gene cloning

Note: M DL 8000 Marker; AaCaM3-cDNA: Cloned using cDNA as template; AaCaM3-gDNA: Cloned cloned using gDNA as template

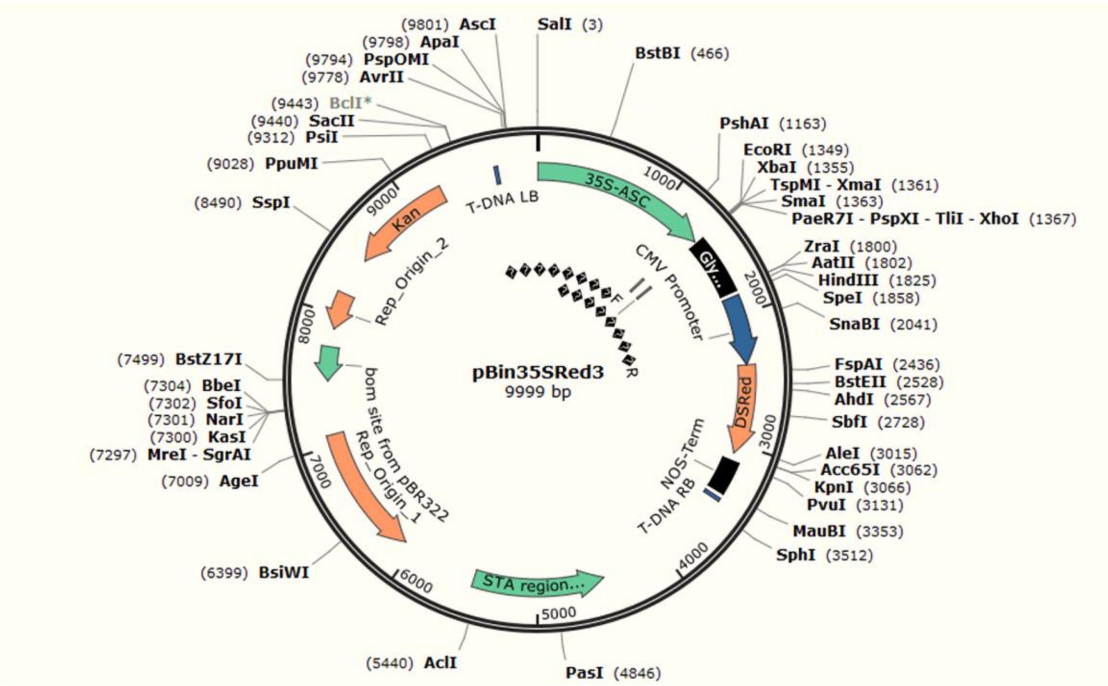

Figure S2 pBin35SRed3 vector used for plant transformation

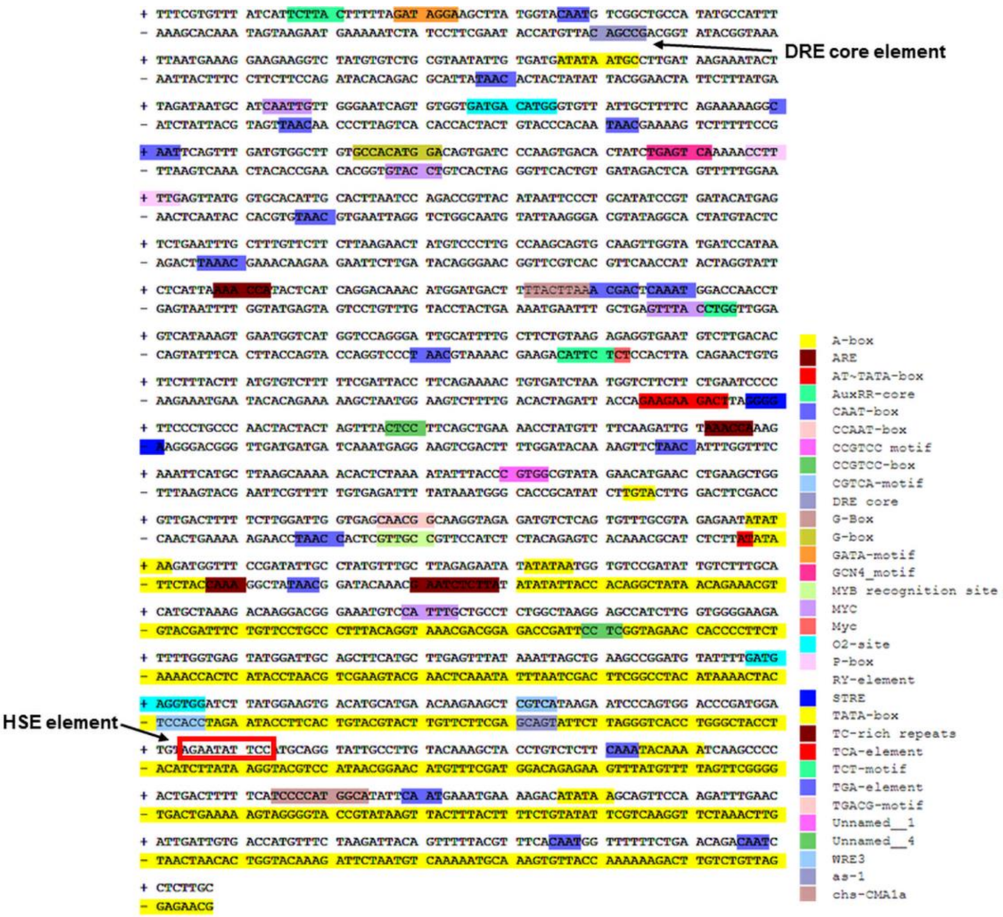

Figure S3 Prediction of *cis*-acting elements on *prAaCaM3* sequence

Note: Key element “HSE element” and “DRE element” are marked with an arrow
