# Supplementary material for: Functional validation of AaCaM3 response to high temperature stress in Amorphophallus albus
Source: BMC Plant Biol. 2024 Jun 28;24:615. doi: 10.1186/s12870-024-05283-2 (PMC11212397; doi:10.1186/s12870-024-05283-2)
Supplement: Supplementary file 2 — Supplementary Material 2 [file 12870_2024_5283_MOESM2_ESM.doc]

Table S1 PCR primers used in this study

|  | Name | Sequence (5’-3’) |
| --- | --- | --- |
| **For cloning** | AaCaM3-F | TACAGTTTTTACGTTTCACAATGGT |
|  | AaCaM3-R | GAATCGGAACTCACATGGTACT |
|  | AaCaM3-SP1 | ATCATCTCATCAACCTCCTCATCCG |
|  | AaCaM3-SP2 | TCCTTCATCTTCCGAGCCATCAG |
|  | AaCaM3-SP3 | GTGATGCAACCTGTGGAGCG |
|  | prAaCaM3-F | TATCGTGTTTATCAATTCTTACCTT |
|  | prAaCaM3-R | TGCAAGAGGATTGTCTGTTCA |
| **For qRT-PCR** | AaCaM3-RT-F | TCTTCGACAAGGACCAGAACG |
|  | AaCaM3-RT-R | AACCTCCTCATCCGTCAACTTC |
|  | AaEIF4A-RT-F | ACAAGATGAGGAGCAGGG |
|  | AaEIF4A-RT-R | GGTGATAAGGACACGAGA |
|  | AtTUB2-RT-F | ATCCGTGAAGAGTACCCAGAT |
|  | AtTUB2-RT-R | AAGAACCATGCACTCATCAGC |
| **For vector construction** | AaCaM3-1300-F | gcTCTAGAATGCTGTGTCCACGTA |
|  | AaCaM3-1300-R | cggGGTACCCTTGGCCATCATAACT |
|  | AaCaM3-pBin35SRed3-F | gcTCTAGAATGCTGTGTCCACGTATT |
|  | AaCaM3-pBin35SRed3-R | ccgCTCGAGTCACTTGGCCATCATAAC |
|  | AaCaM3-AD/BK-F | cgcCATATGATGCTGTGTCCACGTA |
|  | AaCaM3-AD/BK-R | cgcGGATCCTCACTTGGCCATCATA |
|  | prAaCaM3-162-F | tctagaggatccccgGGTACCTTTCGTGTTTATCATTCTTACTTTTTAGA |
|  | prAaCaM3-162-R | atcctctagagtcgaGGCGCGCCTGCAAGAGGATTGTCTGTTCAGA |
|  | prAaCaM3-pAbAi-F | cttgaattcgagctcGGTACCTTTCGTGTTTATCATTCTTACTTTTTAGA |
|  | prAaCaM3-pAbAi-R | atacagagcacatgcCTCGAGTGCAAGAGGATTGTCTGTTCAGA |
|  | prAaCaM3-0800-F | cggGGTACCTTTCGTGTTTATCATTCT |
|  | prAaCaM3-0800-R | cgcGGATCCTGCAAGAGGATTGTCTGT |

**Note：**The lowercase letters in the table are protected bases.
